# Supplementary material for: Comparative genomics provides new insights into the diversity, physiology, and sexuality of the only industrially exploited tremellomycete: Phaffia rhodozyma
Source: BMC Genomics. 2016 Nov 9;17:901. doi: 10.1186/s12864-016-3244-7 (PMC5103461; doi:10.1186/s12864-016-3244-7)
Supplement: Additional file 6: — List of orphan genes with links to PFAM (related to Additional file 1: Table S1). (ZIP 1428 kb) [file 12864_2016_3244_MOESM6_ESM.zip › PFAM_UNIQUE/G00362_P.html]

#                                                                            --- full sequence --- -------------- this domain -------------   hmm coord   ali coord   env coord
  
# target name        accession   tlen query name           accession   qlen   E-value  score  bias   #  of  c-Evalue  i-Evalue  score  bias  from    to  from    to  from    to  acc description of target
  
#------------------- ---------- ----- -------------------- ---------- ----- --------- ------ ----- --- --- --------- --------- ------ ----- ----- ----- ----- ----- ----- ----- ---- ---------------------
  
G00362\_P             -            250 RRM\_1                PF00076.17    70   3.7e-06   25.2   0.1   1   1   9.7e-08   7.4e-06   24.2   0.1     1    63   110   179   110   181 0.85 - RNA recognition motif. (a.k.a. RRM, RBD, or RNP domain)  
G00362\_P             -            250 RRM\_6                PF14259.1     70     1e-07   30.5   0.1   1   1   2.2e-09   1.7e-07   29.8   0.1     1    64   110   180   110   184 0.85 - RNA recognition motif (a.k.a. RRM, RBD, or RNP domain)
